# Autonomic Imbalance in Lymphoma Survivors

**DOI:** 10.3390/jcm10194391

**Published:** 2021-09-26

**Authors:** Keyla Vargas-Román, Jonathan Cortés-Martín, Juan Carlos Sánchez-García, Raquel Rodríguez-Blanque, Emilia Inmaculada De La Fuente-Solana, Lourdes Díaz-Rodríguez

**Affiliations:** 1 Research Group CTS1068, Andalusia Research Plan, Junta de Andalucía, 18014 Granada, Spain; jonathan.cortes.martin@gmail.com (J.C.-M.); jsangar@ugr.es (J.C.S.-G.); raquel.rodriguez.blanque.sspa@juntadeandalucia.es (R.R.-B.); cldiaz@ugr.es (L.D.-R.); 2 Spanish Education Ministry Program FPU16/01437, Methodology of Behavioral Sciences Department, Faculty of Psychology, University of Granada, 18071 Granada, Spain; 3 Department of Nursing, School of Health Sciences, University of Granada, 18016 Granada, Spain; 4 Health Technology Park, San Cecilio University Hospital, 18016 Granada, Spain; 5 Methodology of Behavioral Sciences Department, School of Psychology, University of Granada, 18071 Granada, Spain; edfuente@ugr.es

**Keywords:** lymphoma cancer, HRV, autonomic nervous system, non-Hodgkin lymphoma

## Abstract

Among the types of blood cancers, non-Hodgkin lymphoma is the most common. The usual treatments for this type of cancer can cause heart failure. A descriptive observational study was conducted that included 16 non-Hodgkin lymphoma survivors and 16 healthy controls matched by age and sex. Vagal tone was evaluated in the short term with a three-channel Holter device, and the time and frequency domains were analyzed following a previously accepted methodology to evaluate cardiac autonomic balance. The results of the analysis revealed that the standard deviation of the NN interval (F = 6.25, *p* = 0.021) and the square root of the mean of the sum of the differences between NN intervals (F = 9.74, *p* = 0.004) were significantly higher in healthy subjects than in lymphoma survivors. In the heart rate variability (HRV) index, there were no significant differences between the groups (F = 0.03, *p* = 0.85), nor in the parameters of the frequency domains LF (F = 1.94, *p* = 0.17), HF (F = 0.35, *p* = 0.55), and the ratio LF/HF (F = 3.07, *p* = 0.09). HRV values were lower in non-Hodgkin lymphoma survivors in the first year after treatment, resulting in autonomic imbalance compared to healthy paired subjects.

## 1. Introduction

According to the World Health Organization, cancer is the leading cause of death worldwide and there are around 18.1 million new cases every year, expected to increase to 24 million cases by 2035 [1,2]. Within the World Health Organization’s global hematic cancers, non-Hodgkin lymphoma had 509,590 (2.8%) new cases worldwide in 2018 in both genders. It ranks eleventh in new cases for 2018 [1]. In terms of mortality rate, non-Hodgkin lymphoma cancer caused 248,724 deaths in 2018 in both genders worldwide.

In Spain, in 2020, non-Hodgkin lymphoma was one of the most diagnosed blood cancers (9188), being in the top 10 positions [2]. Worldwide, it is the most frequent, accounting for 3.0% with a mortality rate of 2.6% [3]. Mortality from this tumor type has decreased since the late 1990s at a rate of 3% annually, reflecting a clear improvement in the effectiveness of treatments [2].

The most common treatments for non-Hodgkin lymphoma include chemotherapy, radiation therapy, immunotherapy, and, in certain special cases, bone marrow or stem cell transplants. It is estimated that by 2025, the number of cancer patients that undergo at least one session of radiation therapy will have increased by 16.1% [4]. These therapies produce highly adverse effects such as fatigue, anxiety, depression, insomnia, loss of physical condition, and a high risk of suffering heart failure that leads to a decrease in quality of life [5].

Cancer and cardiovascular disease were previously considered two distinct pathologies. Recent data show that they share multiple risk factors, suggesting that there might be a biological pathway [6]. Patients with Hodgkin lymphoma who have received radiation therapy represent a high-risk group for heart failure, developing arrhythmias, ischemic heart disease, and congestive heart failure [7]. Another study found that in patients undergoing chemotherapy for non-Hodgkin lymphoma, especially with anthracyclines, cardiovascular risk is increased [8]. With increased survival, attention to the adverse effects of treatments received during cancer becomes increasingly important [9].

Heart rate variability (HRV) is a noninvasive biomarker of health that reflects vagal activity and it may be a useful test for autonomic imbalance [10,11]. It results from interaction between the autonomous nervous system and the cardiovascular system. [12] Vagal nerve activity is very strongly correlated with HRV [13] and it is an independent predict prognosis after myocardial infarctions [14] and in cancer. [15] 

No HRV-related studies have been found in patients with non-Hodgkin lymphoma. 

This is why the objective of this descriptive study is to compare, by controlling the confounding variables, the HRV of lymphoma survivors after their first year of treatment completion with those of healthy subjects.

## 2. Materials and Methods

### 2.1. Subjects

A descriptive observational study was conducted that included 16 non-Hodgkin lymphoma survivors and 16 healthy controls matched by age and sex. Short-term vagal tone was evaluated with a three-channel Holter device, and the time and frequency domains were analyzed to evaluate cardiac autonomic balance. The patients were recruited from the oncology department of the Spanish hospital Virgen de las Nieves in Granada, and the healthy controls matched by age and sex were recruited from the general community. Inclusion criteria were a primary diagnosis of non-Hodgkin lymphoma cancer (Grades I-IIIa), age between 18 and 65 years, and the primary part of cancer treatment (surgery, chemotherapy, radiation therapy, and/or immunotherapy) having been completed 6 months to 1 year earlier. Exclusion criteria for this study were the presence of metastasis and/or active cancer, history of cardiovascular disease, and administration of medications known to alter vagus nerve activity. The contact procedure was telephone. Once the patients were cited, the informed consent document was signed, their medical history was completed, a questionnaire was completed for epidemiological data, and the procedure of obtaining HRV was started.

### 2.2. HRV Measurements

The Holter device (NoravHolter DL 800, Braemar, Brunsville, MN, USA) and analysis through NH300 Software (Norav, v. 2.70) were used. The variables that collect the time domain and heart rate were evaluated in the short term. The participants adopted a supine position for 10 minutes in a room with a temperature of 22–25 °C, without external stimulation. ECG signals were acquired using a Holter device for 5 minutes and a modified lead channel II system.

HRV was calculated from ECG records as the time interval between consecutive heartbeats (RR interval). In the time domain, we analyzed the standard deviation of the average from normal to normal interval (NN) (SDNN), the square root of the mean of the differences squared of successive NN intervals (RMSSD), and the number of all NN intervals divided by the maximum of all NN intervals (HRV Index). The main spectral components analyzed in the frequency domain were the low frequency band (LF) (0.04–0.15 Hz), as sympathetic and parasympathetic measure, occupations; high frequency band (HF) (0.15–0.40 Hz), associated with vagal-parasympathetic activity; and the LF/HF ratio, indicating sympathovagal equilibrium. We followed the recommendations from the working group of the European society of cardiology and the American Society of Stimulation and Electrophysiology (Task Force, 1996) [16].

### 2.3. Statistical Analysis

The results were expressed through means with standard deviation for continuous variables and as percentages for categorical variables. A confidence interval of 95% was obtained. Parametric and non-parametric tests were applied after the Shapiro–Wilk test to verify the normal distribution of the data. When necessary, the data were logarithmically transformed to achieve homogeneity of variances. Unidirectional analysis of variance (ANCOVA) was used with the group (healthy and lymphoma) as and between subjects. The variables of heart rate, SDNN, RMSSD, HRV index, HF, LF, and HF/LF ratio were evaluated considering the following covariates: age, studies, marital status, work, smoking, alcohol consumption, menopause, height, weight, and body mass index (BMI). IBM-SPSS 26.0 was used for data analysis, and *p* < 0.05 in the tests was considered statistically significant.

## 3. Results

### 3.1. Demographic and Clinical Data

The study sample of 32 subjects who met the eligibility criteria comprised of 19 females (59.4%) and 13 males (40.6%), the vast majority were Caucasians (96.9%) with a mean (SD) age of 43.13 (7.14) years.

The mean BMI in the group of survivors was 25.81 (5.66), which did not differ significantly from that of the healthy subjects, 24.69 (3.50).

The consumption of tobacco was higher with the healthy group, of which 56.3% of them smoked, contrary to the lymphoma group, 62.6% of which were non-smokers. Of the lymphoma group, 68.8% did not consume alcohol while, in the healthy group, 56.3% did not consume alcohol.

The statistically significant differences between groups were educational level and occupational status, with higher educational levels (68.8%) and more frequent employment (87.5%) in healthy subjects, versus 18.8% and 12.5%, respectively, in the lymphoma survivors group (Table 1).

The dominant type of treatment was chemotherapy, used in 75% of cases. Combination radiation therapy and chemotherapy was used in 6%, and combination immunotherapy and chemotherapy in 19%. The most frequent chemotherapy administered in 80% of cases was a regimen of four drugs known as “CHOP”: cyclophosphamide, doxorubicin, vincristine and prednisone The CHOP treatment plus the monoclonal antibody rituximab, known as “R-CHOP”, was administered in 20% of cases.

Regarding transplants, 87.5% of patients received none, compared to 6% who received autotransplantation and another 6% who received allotransplantation. Among patients, stage VI disease was predominant in 37.5% of cases (Table 2).

#### 3.1.1. HRV Analysis

HRV analysis revealed no significant values between the lymphoma survivors group and the healthy subjects in HRV index (*p* = 0.859). (Table 3).

Significant differences were found between groups in the time domain: RMSSD (F = 9.74, *p* = 0.004) and SDNN (F = 6.25, *p* = 0.018). In the frequency domain parameters, the results were not significant: LF (F = 1.94, *p* = 0.17), HF (F = 0.35, *p* = 0.55), and LF/HF ratio (F = 3.07, *p* = 0.09).

There were statistically significant differences between groups when controlling for covariates like height (RMSSD *p* = 0.001; SDNN *p* = 0.003), weight (RMSSD *p* = 0.021; SDNN *p* = 0.002), BMI (RMSSD *p* = 0.027; SDNN *p* = 0.002), occupational status (RMSSD *p* = 0.045; SDNN *p* = 0.015), and tobacco (RMSSD *p* = 0.029; SDNN *p* = 0.001), and educational level (*p* = 0.000), alcohol (*p* = 0.032), and menopausal status (*p* = 0.002) for SDNN values. In the same way, the type of treatment (RMSSD *p* = 0.041) and the type of medical transplant (RMSSD *p* = 0.047; SDNN *p* = 0.009) influenced HRV parameters. The RMSSD values in combination chemotherapy and immunotherapy treatment were higher (49.08 ± 17.41) than in chemotherapy (26.76 ± 3.27) or a combination of radiation therapy and chemotherapy (23.13 ± 0.00) treatments. The allotransplant patients showed higher scores (SDNN = 54.14 ± 0.00; RMSSD= 52.90 ± 0.00) than non-transplant (SDNN = 38.18 ± 3.73; RMSSD= 29.79 ± 4.63) or autotransplant patients (SDNN = 34.80 ± 0.00; RMSSD= 21.48 ± 0.00).

## 4. Discussion

The main finding in this study was the presence of autonomic imbalance in non-Hodgkin lymphoma survivors during their first year after completing treatment compared to healthy paired subjects, evidenced by the lower values of time domain measures in heart rate variability (SDNN and RMSSD). This imbalance could be related to either the cancer treatment received or remnants of the cancer itself in the survival stage and lifestyle (problems with cholesterol, blood pressure, obesity, among other factors) [17]. Based on the evidence, we estimate that approximately 30 to 50% of deaths caused by cancer could be avoided if risk factors such as tobacco consumption, low dietary consumption of healthy foods such as fruit and vegetables, and excess alcohol consumption, among others, had been modified or prevented [18]. One study found that among breast cancer patients treated with chemotherapy who were obese, there was a relationship between cardiovascular problems and obesity [19]. The influence of these factors is beyond the scope of this study, but deserves further research in the future in order to prevent possible factors that could affect the survival of this population.

In consonance with the evidence presented above, our study found an influence of covariates such as height, weight, BMI, occupational status, tobacco, educational level, alcohol and menopausal status as factors significantly affecting the time domain values comparing non-Hodgkin lymphoma survivors with healthy subjects. This may reinforce the previous evidence stating the risk factors in cancer deaths, Although it has shown the lifestyle, genetic or psychological state influence on HRV [20] mainly HRV reflects the cardiac vagal tone [21]. This could be the significant difference between healthy groups and those with non-Hodgkin lymphoma cancer.

In terms of cancer survival, a study shows how cancer survivors are more likely to have cardiovascular risk factors, such as excess bodyweight and hypertension, compared to healthy patients [22]. Following this line of research, another study found a high incidence of cardiovascular disease in patients with breast cancer, lung cancer, and non-Hodgkin lymphoma compared to the healthy population. Patients with pre-existing cardiovascular problems did worse compared to cancer patients without any heart disease over time [23].

Our findings show a significant difference between groups in terms of time domain in heart rate variability (HRV), thus demonstrating a sympathovagal imbalance. This connects with previous studies, where, in the first year of survival, patients with breast cancer obtained high HRV values [24]. Further research on HRV in cancer patients is needed to establish its potential for clinical follow-up in this population. Based on the results of a previous meta-analysis, HRV is a viable noninvasive tool for assessing prognoses in cancer patients [25].

We recognize that there are some limitations to this study. First, the time taken to measure HRV was 5 min instead of a 24 h observation. However, the Task Force of the Union Society of Cardiology and the American Society of Stimulation and Electrophysiology (1996) recommends the short-term method of measuring HRV as it is the most commonly used non-invasive approach in cancer patients. Second, breathing rhythm was not controlled, suggesting that simultaneous measurement of respiratory rates may provide important additional results in resting HRV [26]. Finally, the small sample size and the uncommon gender (female) and mean age (43) of our population, limits the interpretation and the extrapolation of HRV results. Studies with large samples and a typical mean of age and sex within this population are required to establish references in cancer survivors and determine that the usefulness of HRV in the monitoring of cardiovascular health in patients with non-Hodgkin lymphoma cancer. This would greatly help to use non-pharmacological therapies such as meditation or qigong in combination with traditional medicine to correct the balance of the autonomic nervous system and further improve the prognosis of non-Hodgkin lymphoma survivors [27].

## 5. Conclusions

Thus, the sample size of studied patient is too small to extrapolate a conclusion to a bigger population. Heart rate variability values were lower in non-Hodgkin lymphoma survivors in the first year after treatment than in healthy controls, suggesting a sympathovagal imbalance compared to healthy paired subjects.

## Figures and Tables

**Table 1:** Participant characteristics by study group.

Variable	Healthy Controls (*n* = 16)	Lymphoma Cancer (*n* = 16)	*p* Value
Age (years), mean + SD (range) **	43.13 ± 6.51 (34–56)	43.13 ± 7.94 (32–58)	1
Sex(%) *			0.072
Female	75.0	43.7	
Male	25.0	56.2	
Marital Status (%) *			0.146
Single	0.0	25.0	
Married	75.0	50.0	
Living together	0.0	18.8	
Widowed	6.3	0.0	
Divorced	18.8	6.3	
Educational Level (%) *			0.001 *
Primary Studies	0.0	25.0	
Secondary Studies	31.3	56.3	
High school	68.8	18.8	
Occupational status (%) *			0.008 *
Homemaker	12.5	18.8	
Employed	87.5	12.5	
Sick Leave	0.0	50.0	
Not working due to the disease	0.0	12.5	
Retired	0.0	6.3	
Smoking Status (%) *			0.797
Nonsmoker	18.8	62.5	
Smoker	56.3	12.5	
Ex-smoker	25.0	25	
Alcohol status (%) *			0.325
No consumption	56.3	68.8	
Consume monthly	18.8	25.0	
Consume weekly	25.0	6.3	
Menopausal status (%) *			0.075
No	93.8	68.8	
Yes	6.3	31.3	
Weight (kg), mean + SD (range) **	68.59 ± 15.24 (56–105)	74.84 ± 18.21 (45–108.8)	0.301
Height (cm), mean + SD (range) **	165.75 ± 7.01 (158–179)	169.94 ± 9.18 (150–183)	0.157
BMI (kg/m^2^), mean + SD (range) **	24.69 ± 3.50 (21–32.8)	25.82 ± 5.66 (17.8–40.0)	0.505

Note: Values are expressed as means ± standard deviation (95% confidence interval). Chi-square test * and Student *t*-test ** for between group comparisons; * *p* < 0.05.

**Table 2:** Clinical characteristics of participating non-Hodgkin lymphoma cancer patients.

Variable	Cancer Patients (*n* = 16)
Diagnosis type of lymphoma (%) Non-Hodgkin	16
Tumor Stage (%)	
I	18.8
II	18.8
III	25.0
IV	37.5
Type of medical treatment (%)	
Radiotherapy	0.0
Chemotherapy	75.0
Radiotherapy and Chemotherapy	6.3
Chemotherapy and Immunotherapy	18.8
Type of medical transplant (%)	
Allotransplant	6.3
Autotransplant	6.3
None	87.5

Note: Values are expressed as percentages in the variables referring the lymphoma cancer patients.

**Table 3:** Comparison of the dependent variables of heart rate variability (HRV) between the study groups.

Variable	Healthy Controls (*n* = 16) Mean + SD (95%CI)	Non-Hodgkin Cancer (*n* = 16) Mean + SD (95%CI)	*p* Value
Time domain			
SDNN (ms)	52.63 ± 17.05 (43.54–61.71)	38.97 ± 13.65 (31.69–46.24)	0.018 *
RMSSD (ms)	50.40 ± 18.36 (40.62–60.19)	30.72 ± 17.30 (21.5–39.94)	0.006 *
HRV index	5.81 ± 1.42 (5.05–6.57)	5.73 ± 1.14 (5.12–6.34)	0.859
Frequency domain			
LF (ms)	134.69 ± 41.51 (112.57–156.81)	168.87± 88.70 (121.60–216.14)	0.177
HF (ms)	138.62 ± 0.23 (121.44–155.8)	128.77± 57.49 (98.13–159.4)	0.555
LF/HF ratio	1.04 ± 0.464 (0.794–1.29)	1.36± 0.583 (1.05–1.67)	0.090

Note: ANCOVA for comparisons between interventions * *p* < 0.05, SDNN = standard deviation of the normal-to-normal interval; RMSSD = root mean square of successive differences; LF = low frequency; HF = high frequency; and ANCOVA = analysis of covariance.
